# Inactivation of Enveloped Viruses by a Silver-Thiosulfate Complex

**DOI:** 10.1155/MBD.1994.511

**Published:** 1994

**Authors:** Hiroaki Oka, Toshikazu Tomioka, Katsumi Tomita, Atsushi Nishino, Shigeharu Ueda

**Affiliations:** 1 Matsushita Electric Industrial Co., Ltd. 3-1-1, Yagumo-Nakamachi Osaka Moriguchi 570 Japan; 2 Department of Neurovirology Research Institute for Microbial Diseases Osaka University Yamada-oka Osaka 565 Japan


					INACTIVATION OF ENVELOPED VIRUSES
BY A SILVER-THIOSULFATE COMPLEX
Hiroaki OkaY, Toshikazu Tomioka,Katsumi Tomita,
Atsushi Nishino and Shigeharu Ueda2
Matsushita Electric Industrial Co., Ltd., 3-1-1, Yagumo-Nakamachi, Moriguchi, Osaka 570, Japan
2 Department of Neurovirology, Research Institute for Microbial Diseases, Osaka University,
Yamada-oka, Osaka 565, Japan
AmenitopTM was developed by Matsushita Electric Industrial Co., Ltd. It is an inorganic material
originally developed to prevent bacterial contamination on the surface of appliances such as
telephone and facsimile machines, and consists of silica gel micmspheres containing silver-
thiosulfate complex (AT-l). It has a coating layer of tetraethoxysilanet that enables gradual
release of AT-1 on the surface. AT-1 showed bactericidal effect on various kinds of bacteria such
as E. co S. aumus, methicillin resistant S. aumus (MRSA). So, we examined antiviral effect of
AT-1 on several kinds of viruses such as human immunodeficiency virus-1 (HIV-1, JMH-1), herpes
simplex virus type-2 (HSV-2, G strain), measles virus (MV, Nagahata strain) and polio virus type-
1 (PV, Sabin vaccine type 1).
AT-1 was extracted into phosphate buffered saline (PBS) from AmenitopTM at 37 C.for 30 min to
obtain pure AT-I. The extracted AT-1 was stored at -80 C until use. HIV-1 was propagated in
MT-4 cells. HSV-2, MV and PV were propagated in Veto cells. Stock viruses were prepared from
the culture supematant fluids by low speed centrifugation and stored at -80 C until use.
An aliquot of 500 I1 of the stock virus and AT-1 was mixed and incubated at 37 C, 22 C or 4 C for
a given time. AT-1 was removed from the mixture by centrifugation using Centrisart (Sartorius Co.
Ltd., cut off M.W. 20000) after the incubation. Then, the mixture was supplemented with 1 ml of
PBS. Virus infectivity was measured using microplates by observing cytopathic effect.
AT-1 reduced infectivity of HIV-1103 TCID5o/ml in 30 min at 37 C. The inactivating effect of AT-1
on HIV-1 was temperature-dependent and it failed to inactivate HIV-1 at 4 C. Further, we
examined the inhibiting effect of AT-1 on HIV-1 multiplication. Twelve nM of AT-1 in the culture
medium inhibited multiplication of HIV-1 during 4 to 7 days culture after infection at multiplicity of
infection 0.001 without any cytotoxicity.
Likewise, AT-1 inactivated MV104 TCID5o/ml at 37 C in 30 min. Temperature dependency of the
inactivating effect of AT-1 was the same as in the case of HIV-1 and it failed to inactivate MV at 4
C even after 3 hours incubation. Inactivation of HSV-2 and PV with AT-1 was similarly examined.
AT-1 inactivated HSV-2 106 TCID5o/mI. However, PV did not show any reduction in infectivity.
The results suggest membrane-destroying effect of AT-I.
511

				

